# Strategies to Produce Grapefruit-Like Citrus Varieties With a Low Furanocoumarin Content and Distinctive Flavonoid Profiles

**DOI:** 10.3389/fpls.2021.640512

**Published:** 2021-02-24

**Authors:** A. Garcia-Lor, A. Bermejo, J. Morales, M. Hernández, A. Medina, J. Cuenca, L. Navarro, Pablo Aleza

**Affiliations:** Centro de Citricultura y Producción Vegetal, Instituto Valenciano de Investigaciones Agrarias, Valencia, Spain

**Keywords:** mandarin, pummelo, citrus, antioxidants, 6,7-dihydroxybergamottin, naringin, breeding, triploids

## Abstract

Pummelos and hybrids, such as grapefruits, have high furanocoumarin and low flavonoid contents. Furanocoumarins interact negatively with certain drugs, while flavonoids are antioxidant compounds with health benefits. To obtain new grapefruit-like varieties with low furanocoumarin and high flavonoid contents, diploid and triploid hybrid populations from crosses between diploid and tetraploid “Clemenules” clementine and diploid “Pink” pummelo were recovered and analyzed. With regard to furanocoumarins, triploids produce less bergapten, bergamottin and 6,7-DHB than diploids. Regarding flavonoids, triploids yielded more eriocitrin, narirutin, hesperidin and neohesperidin than diploids, whereas no differences were observed in neoeriocitrin and naringin. These results indicate that, the strategy to recover triploid hybrids by 4x × 2x crosses is more appropriate than the recovery of diploid hybrids by 2x × 2x crosses for obtaining grapefruit-like varieties of citrus with lower furanocoumarin and higher flavonoid contents.

## Introduction

Citrus fruits are characterized by their distinctive flavor and are considered functional foods, due to their antioxidant capacity as well as their nutrient content. They are a good source of vitamin C, carbohydrates, dietary fiber, many B vitamins, minerals, and biologically active phytochemicals such as carotenoids and flavonoids (Liu et al., 2012). Phenolic compounds or polyphenols such as flavonoids are bioactive substances that occur widely in fruits, vegetables, and cereal grains and are strongly associated with the sensory and nutritional qualities of fresh and processed plant foods. Flavonoids have six different classes, including flavanones, flavonols, flavones, isoflavones, anthocyanidins, and flavanols. Citrus fruits are recognized as a main source of flavanones which affect fruit flavor and have been recognized to improve human health (Abbas et al., 2017). Hesperidin, neohesperidin, narirutin, eriocitrin, neoeriocitrin, and naringin are the most abundant flavanones in citrus fruits, although differences in their contents are present on citrus species and its hybrids. Hesperidin is the most abundant flavanone in sweet oranges [*Citrus sinensis* (L.) Osb.], mandarins (*C. reticulata* Blanco), and tangors (*C. reticulata*× *C. sinensis*). Naringin and narirutin are present in greater quantities in grapefruit (*C. paradisi* Macf.) and neohesperidin and naringin are particularly abundant in tangelos (*C. reticulata*× *C. paradisi*) (Peterson et al., 2006a, b). Naringin is responsible for the bitter taste of grapefruit, and this flavanone together with hesperidin, are recognized as a major bioactive components responsible for the anticancer activities of grapefruit (Hung et al., 2017). Neoeriocitrin, naringin and neohesperidin are potential therapeutic agents to protect cartilage tissue (Crasci and Panico, 2013). Therefore, the development of new citrus varieties with a high flavonoid content would allow commercialization as improved varieties related to nutrition and prevention of diseases and would also allow the promotion of citrus breeding programs in a field with wide future possibilities of innovation.

Genetic breeding and molecular strategies have been used to exploit flavonoid biodiversity present among plant species (D’amelia et al., 2018). Although it is challenging to improve characteristics controlled by several genes and environmental conditions with sexual hybridization (Goulet et al., 2017), it has been achieved in other species like tomato (Willits et al., 2005), where quercetin production was enhanced using a breeding approach utilizing the genetic diversity available in wild germplasm resources, or maize where flavonoid composition was improved (Petroni et al., 2014).

Coumarins and furanocoumarins (FCs), widely distributed in plants, especially in the families *Apiaceae*, *Rutaceae*, *Moraceae*, and *Fabaceae*, are involved in plant defense against pathogens and in environmental adaptation (Bourgaud et al., 2006). Within the *Citrus* genus, which belongs to the *Rutaceae* family, the pummelos [*C. maxima* (Burm.) Merr.] and their hybrids, such as grapefruits and sour oranges (*C. aurantium* L.), present high amounts of FCs in the pulp whereas mandarins and sweet oranges present the lowest FC content (Dugrand-Judek et al., 2015). It has been demonstrated that FCs increase the bioavailability of certain medications (Bailey et al., 2013), which is called the grapefruit juice effect (GJE). This is due to the interaction between FCs and cytochrome P450 (CYP3A4) in the human intestine, provoking their inhibition and the consequent increase in the level of some drugs in blood, which can cause severe alterations in the cardiac rhythm or respiratory depression (Bailey et al., 2013), or severe phytophotodermatitis after skin contact followed by UV exposure from the sun (Robl et al., 2013), among other adverse effects. The FCs that present a strong interaction, ranked in descending order, are paradisin C, 6,7-dihydroxybergamottin (6,7-DHB), bergamottin, isoimperatorin, bergapten, and bergaptol (Chen et al., 2011), being 6,7-DHB the highest accumulated in pummelo and its hybrids (Chen et al., 2011; Dugrand-Judek et al., 2015). Consequently, FCs have an adverse influence on the marketing and consumption of grapefruit fruit and juice. To remove these undesirable effects from processed juice, different methods have been developed, such as different extraction processes and heat and UV radiation treatments. However, these treatments require additional costs and may reduce grapefruit juice quality (Uesawa and Mohri, 2006a, b). Despite their FCs, grapefruit fruits and juice contain other phytochemicals that have been proven to exhibit beneficial properties for human health (Hung et al., 2017). However, the consumption of grapefruit fruit and juice has dropped considerably since the interference of certain compounds with drug absorption was identified. For this reason, it would be highly desirable to obtain grapefruit-like citrus varieties with low or no FC content.

Some studies have shown that several genes are involved in the production of FCs in plants (Larbat et al., 2007; Vialart et al., 2012; Chen et al., 2014). Three potent FCs were quantitatively correlated in most varieties and hybrids, and the corresponding trait, likely segregating in a Mendelian manner, appeared to be controlled by a single regulatory or enzymatic gene in the FC biosynthesis pathway (Chen et al., 2011). Subsequently, Contig 6 was found to be related to reduced FC levels in the mutant Low Acid Foster grapefruit (Chen et al., 2014).

Grapefruit is a natural hybrid between pummelo and sweet orange of relatively recent origin (Nicolosi et al., 2000; Garcia-Lor et al., 2013). In fact, the earliest mention of grapefruit was in 1750 on the Caribbean island Barbados. Grapefruits have been diversified by spontaneous mutations (Barry et al., 2020), display high heterozygosity and have very low genetic variability (Corazza-Nunes et al., 2002). Therefore, it is not possible to carry out genetic improvement within the species. To overcome this problem, sexual hybridization could be a useful strategy, but would not recover true-to-type grapefruit varieties; however, this strategy could be suitable for the production of new grapefruit-like varieties with low FCs content and distinctive flavonoid profiles, together with other characteristics attractive to the consumers, as shape and size of the fruit, color of the peel and pulp or seedlessness. This strategy, at diploid and triploid levels, is one of the most promising approaches and in fact has been used in breeding programs in Florida, Spain, Israel, and Italy (Grosser et al., 2015; Navarro et al., 2015; Fidel et al., 2016; Russo et al., 2016), respectively. Citrus diploid hybrids can be recovered by classical sexual hybridization between diploid parents, whereas triploid hybrids can be produced directly from crosses between two diploid genotypes resulting from the union of a 2n megagametophyte with haploid pollen (Aleza et al., 2010) or by hybridization between diploid and tetraploid parents (Aleza et al., 2012a, b). Seedlessness is one of the most important characteristics of citrus on the fresh fruit market since consumers do not accept seedy fruits. The creation of triploid hybrids is an important breeding strategy to develop new commercial varieties of seedless citrus (Ollitrault et al., 2008; Navarro et al., 2015). Triploid plants are generally considered an evolutionarily dead-end since they generally give rise to aneuploid gametes with very low fertility (Otto and Whitton, 2000). For these reasons, citrus triploid hybrids are generally sterile. In 1995, we started a citrus breeding program mainly focused on mandarins, although attention was also given to the development of new grapefruit-like varieties. In this context, populations of diploid and triploid hybrids were obtained using diploid and tetraploid “Clemenules” clementines (*C. clementina* Hort. ex Tan.) as the female monoembryonic parents and “Pink” pummelo as male parent. “Clemenules,” a natural hybrid between mandarin and sweet orange, is one of the most important mandarin varieties widely cultivated in the Mediterranean Basin, especially in Spain, due to its excellent organoleptic properties and lack of FCs, while “Pink” pummelo has red-fleshed pulp when grown under appropriate environmental conditions, good flavor, and a high FC content (Dugrand-Judek et al., 2015).

The purpose of our research was to study how the ploidy level of the female parent affects the accumulation of FCs and flavanones in diploid and triploid hybrids to identify the best strategy to recover grapefruit-like citrus hybrids with low FC and high in flavanone contents.

## Materials and Methods

### Plant Materials

We used two strategies to obtain grapefruit-like hybrids; diploid hybrids were recovered from 2x × 2x sexual hybridization, and triploid hybrids were recovered from 4x × 2x sexual hybridization. We used diploid and tetraploid “Clemenules” clementines as female monoembryonic and self-incompatible parents (hereafter Clem 2x and Clem 4x, respectively) and “Pink” pummelo as male parent (hereafter Pum 2x). Clem 4x was obtained by shoot tip grafting *in vitro* combined with colchicine treatment (Aleza et al., 2009). Practical details on the recovery of diploid and triploid hybrids from sexual hybridizations followed by embryo rescue and flow cytometry can be found in Aleza et al. (2010, 2012b), respectively. From these two populations, 116 diploid and 91 triploid hybrids were recovered and grafted onto “Carrizo” citrange rootstock (*C. sinensis* × *P. trifoliata*) in 2011 and 2007 respectively, for field evaluation at IVIA plots. Fifteen diploid and 15 triploid hybrids (numbers 1–15 for diploids and 16–30 for triploids along the text) were selected randomly to perform this study. Fruit samples were taken at three harvest times (December, January, and February) and in three consecutive seasons, 2015/2016, 2016/2017, and 2017/2018.

In parallel, four diploid grapefruits, “Flame,” “Rio Red,” “Star Ruby,” and “Duncan,” in addition to their corresponding tetraploid genotypes, were analyzed to study the effect of ploidy level on FC and flavonoid compounds. Tetraploid grapefruits were obtained from spontaneous chromosome doubling of nucellar cells (Aleza et al., 2009).

### Genotyping

Selected diploid and triploid hybrids together with their parents were analyzed with six simple sequence repeat (SSR) markers [TAA41 (Kijas et al., 1997), MEST56 (Aleza et al., 2009), mCrCIR07B05 and Ci07C09 (Froelicher et al., 2008), mCrCIR02F12 and mCrCIR01C06 (Cuenca et al., 2011)], displaying heterozygosity and polymorphism between parents (A_1_A_2_ × A_3_A_4_). The genetic configuration and allele dosage of diploid and triploid hybrids were inferred directly from electroferograms.

Genomic DNA was isolated using a Plant DNeasy kit from Qiagen Inc. (Valencia, CA, United States) following the manufacturer’s protocol. PCR conditions and data analysis were as reported by Garcia-Lor et al. (2012). Denaturation and capillary electrophoresis were carried out on a Capillary Gel Electrophoresis CEQTM 8000 Genetic Analysis System using linear polyacrylamide according to the manufacturer’s instructions (Beckman Coulter Inc.). Genetic analysis system software (GenomeLabTM GeXP version 10.0) was used for data collection and analysis. Alleles were sized based on a DNA size standard (400 bp).

### Sample Preparation, Chromatography (HPLC), and Identification of Compounds

The main flavonoids and FCs were extracted and determined according to the procedure described by Cano et al. (2008) with some modifications to adapt the method to a microliter format. Three fruits from each hybrid were selected for juice extraction with a mechanical press. Three technical replicates were taken. Juice was frozen at −20°C until it was processed. Briefly, 1 mL of filtered juice was mixed with 1 mL of DMSO/MeOH (1:1, v/v). Then the sample was centrifuged (Eppendorf 5810R centrifuge; Eppendorf Iberica, Madrid, Spain) at 4°C for 15 min at 8,000 × g. The supernatant was filtered through a 0.45 μm nylon filter and analyzed by HPLC-DAD and HPLC-MS in a C18 reversed-phase column [Waters, Nova-Pak C18 4 μm (3.9 × 150 mm)]. An analysis was performed using an Alliance liquid chromatographic system (Waters, Barcelona, Spain) equipped with a 2,695 separation module coupled to a 2,996 photodiode array detector and a ZQ2000 mass detector. The gradient mobile phase consisted of acetonitrile (solvent A), H_2_O (solvent B), and 0.1% acetic acid (solvent C). The flow rate was 0.75 mL min^–1^, and the injection volume was 5 μL. Empower 2 software was used for data acquisition. The sample and column temperatures were 5 and 25°C respectively, and the UV–Vis spectra were recorded from 210 to 500 nm.

HPLC-MS analysis was performed under electrospray ion positive conditions. The capillary voltage was 3.50 kV, cone voltage was 20 V, source temperature was 100°C, desolvation temperature was 225°C, cone gas flow rate was 70 L⋅h^–1^ and desolvation gas flow rate was 500 L⋅h^–1^. Full data acquisition was performed by scanning 150–1,000 uma in centroid mode.

Compounds were identified based on comparison of their retention times, UV-Vis spectra and mass spectrum data with those of authentic standards using external calibration curves (Table 1). Bergamottin and bergapten were purchased from Fluka (Sigma-Aldrich); eriocitrin, neoeriocitrin and narirutin were purchased from Extrasynthesis (Genay, France); and naringin, hesperidin and neohesperidin were purchased from Sigma (Sigma-Aldrich, Barcelona, Spain). The compound 6,7-dihydroxybergamottin (6,7-DHB), was identified tentatively based on the retention times, spectroscopic data and characteristics as described in the literature (Manthey and Buslig, 2005), and its concentration was determined using an external calibration curve with the available standard bergamottin purchased from Extrasynthesis, Genay, France.

**TABLE 1:** Chromatographic, spectral characteristics and mass data of furanocoumarins and flavonoids found in the citrus fruits analyzed.

Compounds	r_*T*_*^b^* (min)	[M + H]^+^ (*m/z*)	λmax (nm)
**Furanocoumarins**			
Bergapten*^a^*	17.68	217	222/267/311
6,7-Dihidroxybergamottin*^c^*	18.83	373	222/250/310
Bergamottin*^a^*	28.98	339	222/250/309
**Flavonoids**			
Eriocitrin*^a^*	7.41	597	227.0/283.6
Neoeriocitrin*^a^*	7.86	597	227.0/283.6
Narirutin*^a^*	8.76	581	227.0/283.6/331.2
Naringin*^a^*	9.22	581	227.0/283.6/331.2
Hesperidin*^a^*	9.52	611	227.0/283.6/330
Neohesperidin*^a^*	9.96	611	227.0/283.6/330

*^*a*^Identified using authentic standards. ^*b*^r_*T:*_ retention time. ^*c*^Tentative identification by comparison with data available in the literature, Manthey and Buslig (2005).*

### Phenotypic Data Analysis

A factorial analysis was performed for the phenotypic data using DARwin5 (Dissimilarity Analysis and Representation for Windows) software version 5.0.159 (Perrier and Jacquemoud-Collet, 2006). For the analysis of the FC and flavonoid contents, the complete dataset of all replicates from the three collection dates of the fully completed season 2015/2016 was used. Single data file format was used as DARwin input, where each unit was characterized by a single value for each variable. The dissimilarity matrix was calculated using the usual Euclidean index for continuous data. This method works on distance matrices, considering the high dimension space defined by the distances between two units. Factorial methods aim mainly to give an overall representation of diversity and do not truly reflect individual effects.

### Statistical Analyses

Statistical procedures were performed using Statgraphics plus 5.1 software (Manugistics, Inc., Rockville, MD, United States). All data were subjected to analysis of variance (ANOVA), and means were compared using the LSD test at *P* < 0.05.

## Results and Discussion

### Genotyping of Diploid and Triploid Hybrids

**FIGURE 1:**
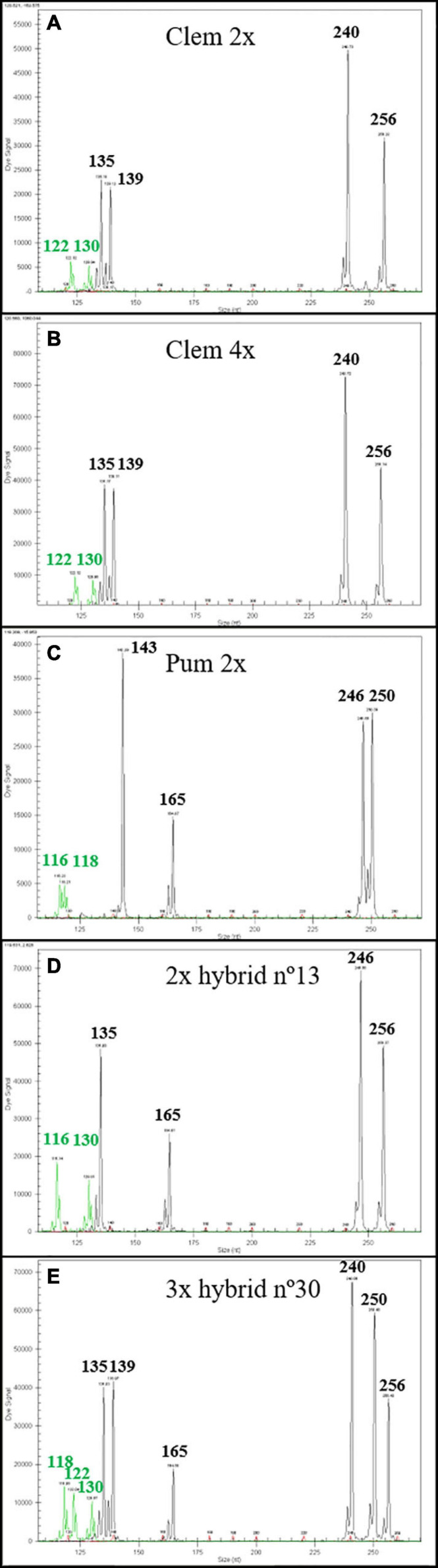
Genetic analysis with mCrCIR02F12, MEST56, and Ci07C09 SSR markers of one diploid and one triploid hybrids with diploid and tetraploid “Clemenules” clementine as female parents and “Pink” pummelo as male parent, respectively. The size of each allele is in nucleotides (nt). **(A)** Diploid “Clemenules” clementine. **(B)** Tetraploid “Clemenules” clementine. **(C)** Diploid “Pink” pummelo. **(D)** Diploid hybrid n°13 recovered from Clem 2× × Pum 2× sexual hybridization. **(E)** Triploid hybrid n°30 recovered from Clem 4× × Pum 2× sexual hybridization.

Genetic analysis of 15 diploid and 15 triploid hybrids with six SSR markers confirmed that diploid hybrids were produced by haploid clementine female gamete with haploid pummelo male gamete, whereas triploid hybrids have been originated by the fecundation of a diploid female gamete from tetraploid clementine with haploid pummelo gamete, as expected, confirming the double mandarin genetic dosage of the triploid hybrids. Figure 1 shows diploid hybrid number 13 with one allele from the female parent and the other allele from the male parent (Figure 1D). In the case of triploid hybrid number 30 (Figure 1E), we observed two alleles from the tetraploid female parent and one allele from the male parent.

### Quantification of Furanocoumarins

In Table 2, we summarized the concentration of each FC in diploid and triploid hybrids and their parents for each harvest time and across the three seasons. Among the three FCs analyzed, 6,7-DHB was present at higher quantities at all harvest times and across the three seasons than were bergapten and bergamottin.

**TABLE 2:** Average furanocoumarin concentration (mg/L) in the diploid and triploid hybrids analyzed and their parents for three harvesting times in three consecutive seasons.

		Bergapten			6,7-DHB			Bergamottin		
Average value for	Samples	S1	S2	S3	S1	S2	S3	S1	S2	S3
HT1	2x	2.37*	0.87*	2.08	16.12*	13.63*	21.84*	1.64*	1.49	1.17
	3x	1.27	0.50	1.50	7.48	5.56	14.55	1.19	1.43	0.75
HT2	2x	1.37*	0.88*	1.53	15.59*	14.46*	16.53	1.64*	1.46	1.13*
	3x	0.58	0.48	1.50	5.90	8.77	14.26	1.17	1.38	0.62
HT3	2x	1.24*	0.50	2.03	12.83*	16.19*	16.84*	1.15*	1.41^*^	0.81*
	3x	0.65	0.80	1.29	8.69	7.91	9.35	0.27	1.80	0.26
The three HT	2x	1.66*	0.75*	1.88*	14.85*	14.76*	18.40*	1.48*	1.45*	1.04*
	3x	**0.83**	**0.59**	**1.43**	**7.36**	**7.41**	**12.72**	**0.88**	**1.53**	**0.54**
	Clem 2x	0.00*^a^*	0.00^a^*	0.20*^a^*	2.02*^a^*	0.00^a^*	6.74*^a^*	0.00*^a^*	0.00^a^*	0.77*^a^*
	Clem 4x	0.00*^a^*	0.00^a^*	0.13*^a^*	3.47*^a^*	0.00^a^*	2.04*^a^*	0.00*^a^*	0.00^a^*	0.00*^b^*
	Pum 2x	2.51*^b^*	4.97*^b^*	2.80*^b^*	43.47*^b^*	68.83*^b^*	33.18*^b^*	1.56*^b^*	2.03*^b^*	2.13*^c^*

S1, Season 2015/2016; S2, Season 2016/2017; S3, Season 2017/2018; HT1, Harvest time 1, December; HT2, Harvest time 2, January; HT3, Harvest time 3, February. Numbers indicate the average value of the 15 diploid or triploid hybrids analyzed in each season and at each harvest time. Bold numbers indicate the average value of the 15 diploid or triploid hybrids and their parents analyzed during the three harvest dates for each season. *Significant differences (p < 0.005) between diploid and triploid hybrids for each compound. ^*a,b,c*^Significant differences (p < 0.005) between Clem 2x, Clem 4x, and Pum 2x. ^a^*Value 0.00 indicates compound not detected, it was obtained only in HT1, in the other two HT no fruit was collected.

A great difference was observed between parents, as expected according to Dugrand-Judek et al. (2015). Pum 2x produced much more FCs than Clem 2x or Clem 4x and these differences were particularly notable for the 6,7-DHB content. It is important to note that the change in “Clemenules” clementine ploidy level from 2x to 4x did not significantly modify the FC content. Clem 2x and Clem 4x did not accumulate bergapten and bergamottin in the S1 and S2 seasons, and extremely low amounts were recorded in the S3 season. In the case of 6,7-DHB, a very low amount was observed in S1 and S3 seasons (Table 2).

**FIGURE 2:**
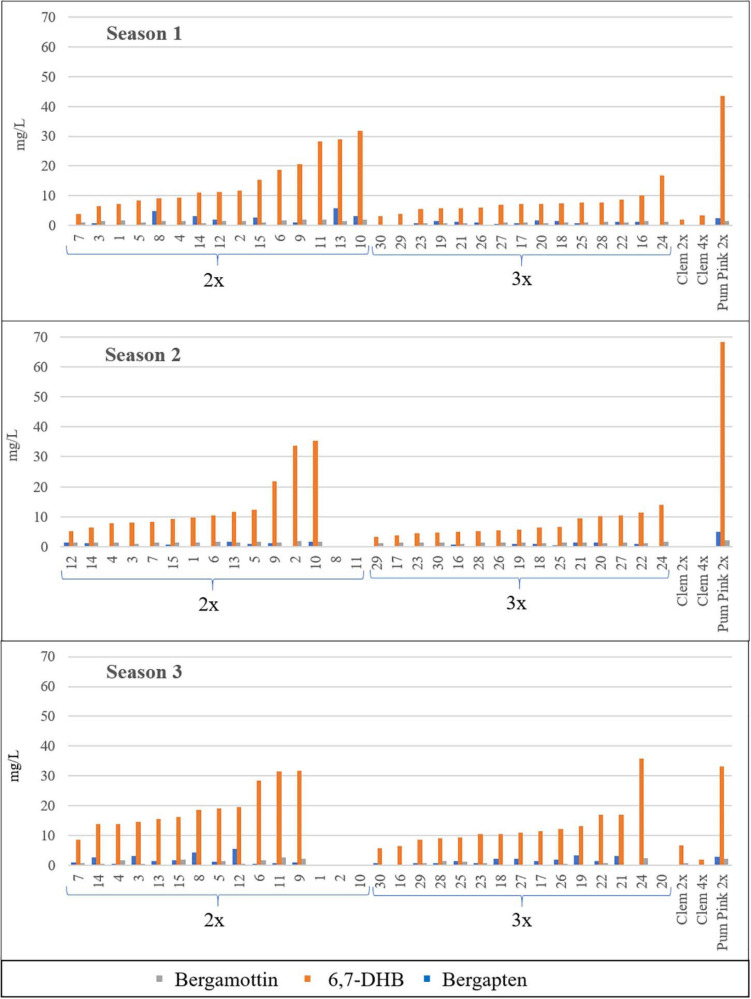
Furanocoumarin contents (mg/L) of all the diploid (2x) and triploid (3x) hybrids and their parents in the three seasons analyzed. Average value of the three harvest times represented. No value indicates no fruit; therefore, FCs were not quantified. Diploid and triploid hybrids were ranked in ascending order according to their total furanocoumarin content.

FC concentrations varied between 2x and 3x hybrids (Table 2). For all FCs, harvest times and seasons, the average FC content in triploid hybrids was lower than that in diploid hybrids, with the unique exception of bergamottin in the 2016/2017 season, although this difference was very small. For example, the average value for all diploid and triploid hybrids along the three harvest times, triploid hybrids produced 21% (Bergapten, S2), 48% (Bergamottin, S3), and 50% (Bergapten, S1; 6,7-DHB, S1 and S2) fewer FCs than diploid hybrids in three consecutive seasons. Diploid hybrids produced 1.66 mg/L bergapten in 2015/2016, whereas triploid hybrids yielded 0.83 mg/L, 50% less, being the greatest difference between the two populations. In S2 and S3 this difference decreased till 1.27- and 1.31-fold, respectively. In the same way, diploid hybrids produced 14.85 mg/L 6,7-DHB in S1, whereas triploid hybrids yielded 7.36 mg/L 6,7-DHB (2.02-fold more content). In S2 and S3 this difference reached 1.99- and 1.45-fold, respectively. Regarding bergamottin, the greatest difference was also observed in the same season, when diploid hybrids produced 1.48 mg/L, whereas triploid hybrids produced 0.88 mg/L, 1.68-fold more. In S3, diploids had 1.93-fold more than triploids, but in S2 a very low difference was observed (1.45/1.53 mg/L; 0.95-fold content). Figure 2 shows the range of FC accumulation in each diploid and triploid hybrid compared with their parents across the three seasons, with triploid hybrids having on average a lower amount of FCs than the diploid hybrids and the male parent Pum 2x but a higher amount than the female parents (Clem 2x and Clem 4x). Nevertheless, at the individual level, some triploid hybrids produced more FCs than some diploid hybrids. For example, if we compare the FC content of triploid hybrid number 24, it always produced more FCs than other diploid hybrids. This could be associated with the recombination of a particular genetic configuration (Schnable et al., 1998) or the maternal imprinting (Ahmed et al., 2020) that favors greater FC production. However, at the population level, triploid hybrids produce less FC content than diploid hybrids, as it was observed by Chen et al. (2011).

The FC content was measured in three different months (December, January, and February) during three different seasons (2015/2016, 2016/2017, and 2017/2018). In the average values for 2x and 3x hybrids, differences were observed between months for each season, although these differences were not pronounced. Nevertheless, the main differences were observed between seasons, and the highest FC accumulation was observed during the season 3. For example, diploid hybrid number 13 reached a total FC content of 36.24 mg/L in the season 3, whereas it produced, 14.76 and 17.36 mg/L in the season 2 and season 1, respectively (Figure 2). Chen et al. (2011) also indicated changes in the FC contents in citrus hybrids depending on the harvest times. For this reason, it is very important to evaluate the FC content in different seasons to minimize this variation. According to the IVIA weather station (Supplementary Figure S1), the highest number of chilling hours (defined as hours with temperature under 7°C) was recorded in the third season analyzed during the months of December 2017 and January and February 2018. The abiotic stress induced by cold temperatures could be associated with the higher FC contents of citrus fruits, as happens in leaves of *Bituminaria bituminosa* (Walker et al., 2012), in which FC levels are higher in winter or in *H. lanatum* where FC levels increased after exposure to cold autumnal nights (Zobel and Brown, 1990). However, although environmental conditions clearly influence the FC content, the genetic background of hybrids seems to be a key factor in the development of new grapefruit-like varieties with a low FC content.

### Quantification of Flavonoids

Table 3 summarizes the concentrations of each flavonoid in the diploid and triploid hybrids and their parents for each harvest time and for the three seasons. As with the FCs, there was huge variability among the flavonoids, and some significant differences were observed. Between parents, significant differences were observed for naringin, narirutin, and hesperidin across the three seasons analyzed. Pum 2x produced much more naringin than Clem 2x and 4x, and in contrast, clementines produced more narirutin and hesperidin than pummelo. These differences agree with previous results (Peterson et al., 2006a, b; Gatusso et al., 2007). For the other compounds (eriocitrin, neoeriocitrin, and neohesperidin), significant differences were also observed between parents in some seasons but not in all three seasons, as was the case in the compounds discussed above. Regarding Clem 2x and 4x, the average values obtained for all compounds were highly variable, and there were no significant differences for all three seasons, although differences were observed in one or two seasons.

**TABLE 3:** Average flavonoid concentrations (mg/L) in the diploid and triploid hybrids analyzed and their parents at three harvest times in three consecutive seasons.

		Eriocitrin			Neoeriocitrin			Narirutin			Naringin			Hesperidin			Neohesperidin		
Average value for	Samples	S1	S2	S3	S1	S2	S3	S1	S2	S3	S1	S2	S3	S1	S2	S3	S1	S2	S3
HT1	2x	5.60*	6.84	8.63*	27.63	26.57	45.37*	14.40*	15.41*	20.74	68.10	60.17	81.82	1.81*	1.71*	4.64*	26.26*	19.55*	19.88*
	3x	7.44	7.62	6.47	24.25	22.00	12.73	23.39	22.00	24.86	55.51	61.33	90.47	11.76	18.83	19.09	49.32	75.48	53.53
HT2	2x	5.64*	7.87	4.34*	27.49	41.18	13.38	12.56*	17.04*	11.10*	61.92	85.17	38.91	2.02*	2.39*	3.82*	25.44*	28.17*	12.42*
	3x	8.98	9.34	5.75	31.54	32.46	11.48	21.61	26.84	19.12	65.16	90.76	48.82	12.26	15.24	11.71	59.18	66.89	46.30
HT3	2x	6.22*	8.18*	4.79*	25.72	35.37	16.03	14.22*	19.81*	11.87*	69.17	110.52*	71.95	1.52*	1.72*	4.57*	25.55	27.86*	18.17*
	3x	8.14	11.64	6.63	29.60	50.83	14.21	22.81	27.01	20.13	61.75	74.57	57.82	11.91	15.81	12.78	59.57	56.40	50.11
The three HT	2x	5.82*	7.63*	5.92*	**26.95**	**34.37**	**24.93**	13.72*	17.42*	14.57*	**66.39**	**85.29**	**64.23**	1.78*	1.94*	4.34*	25.75*	25.19*	16.82*
	3x	**8.19**	**9.54**	**6.28**	**28.46**	**35.09**	**12.81**	**22.60**	**25.28**	**21.37**	**60.80**	**75.56**	**65.70**	**11.98**	**16.63**	**14.52**	**56.02**	**66.26**	**49.98**
	Clem 2x	1.90*^a^*	1.60*^a^*	2.40*^a^*	4.84*^a^*	4.87*^a^*	8.90*^a^*	10.84*^a^*	5.27*^a^*	11.89*^a^*	5.56*^a^*	0.00^a^*	41.03*^a^*	166.71*^a^*	122.87*^a^*	192.30*^a^*	9.42*^a^*	0.00^a^*	6.03*^a^*
	Clem 4x	1.89*^a^*	1.27*^b^*	0.71*^b^*	5.31*^a^*	4.40*^a^*	2.48*^b^*	24.36*^b^*	6.33*^b^*	15.23*^a^*	20.89*^a^*	0.00^a^*	4.40*^b^*	269.13*^b^*	153.73*^b^*	206.53*^a^*	37.44*^b^*	0.00^a^*	3.76*^a^*
	Pum 2x	0.00*^b^*	0.00*^c^*	3.47*^a^*	0.00*^b^*	0.00*^b^*	4.47*^b^*	0.29*^c^*	0.00*^c^*	2.40*^b^*	132.13*^b^*	88.44*^b^*	108.73*^c^*	15.27*^c^*	0.00*^c^*	11.36*^b^*	1.91*^c^*	0.16*^a^*	1.96*^a^*

S1, Season 2015/2016; S2, Season 2016/2017; S3, Season 2017/2018; HT1, Harvest time 1, December; HT2, Harvest time 2, January; HT3, Harvest time 3, February. Numbers indicate the average value of the 15 diploid or triploid hybrids analyzed in each season and at each harvest time. Bold numbers indicate the average value of the 15 diploid or triploid hybrids and their parents analyzed during the three harvest dates for each season. *Significant differences (p < 0.005) between diploid and triploid hybrids for each compound. ^*a,b,c*^Significant differences (p < 0.005) between Clem 2x, Clem 4x, and Pum 2x. ^a^*Value 0.00 indicates compound not detected, it was obtained only in HT1, in the other two HTs no fruits were collected.

**FIGURE 3:**
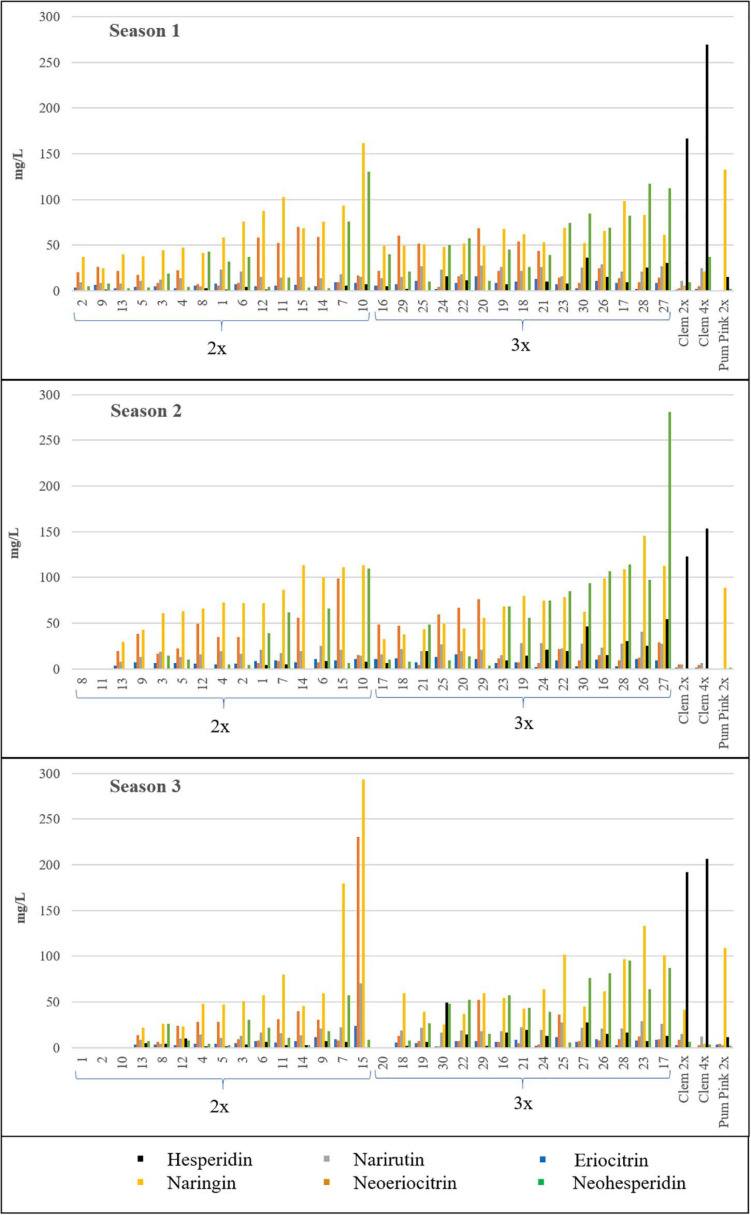
Flavonoid contents (mg/L) of all the diploid (2x) and triploid (3x) hybrids and their parents in the three seasons analyzed. Average value of the three harvest times represented. No value indicates no fruit; therefore, flavonoids were not quantified. Diploid and triploid hybrids have been ranked in ascending order according to their total flavonoid content.

Naringin was identified in the highest quantities in both types of hybrids, although no significant differences were observed between them, as also occurred for neoeriocitrin. However, hesperidin, which was the dominant compound in Clem 2x and 4x, was produced in significantly higher amounts in triploid hybrids than in diploid hybrids (6.73-fold more content in S1, 8.57-fold in S2 and 3.35-fold in S3). In addition, eriocitrin, narirutin, hesperidin, and neohesperidin contents were also significantly higher in triploid hybrids than in diploid hybrids (Table 3). As an example, triploid hybrids produced between 3 and 9-fold more hesperidin than diploid hybrids (14.52–4.34 mg/L at S3 and 11.98–1.78 mg/L at S1), whereas the differences were slightly smaller for neohesperidin, with triploid hybrids producing between two to three times more neohesperidin than diploid hybrids for all seasons, specifically 2.18-, 2.63-, and 2.97-fold more content in S1, S2, and S3, respectively (Table 3). Figure 3 shows the range of flavonoid accumulation in each diploid and triploid hybrid compared with their parents across the three seasons. There was great variation between hybrids at the same ploidy level and even between seasons. The flavonoid profiles for each diploid and triploid hybrid were specific and complex. For example, triploid hybrid number 17 yielded the maximum total flavonoid compounds in season 1 and season 3, producing 234.30 and 245.59 mg/L, respectively, whereas in season 2, 124.04 mg/L was produced, considerably less than in the other two seasons. For the average values for 2x and 3x hybrids, differences were observed between harvest times for each season, although they were not generally pronounced, with minor exceptions. Nevertheless, the main differences were observed between seasons, and the highest flavonoid accumulation was observed during season 2. Such results suggest that flavonoid production could be influenced by environmental conditions, as we have also noted for FC contents. Numerous studies have been published about the influence of environmental factors on the biosynthesis of flavonoids (Jaakola and Hohtola, 2010; Wu et al., 2019; Morales et al., 2020; Przybylska-Balcerek et al., 2020; Morales et al., 2021). Jaakola and Hohtola (2010) indicated the effect of high or low temperature on the composition or concentrations of flavonoids in many plant species. The nutritional composition of mandarin fruits, particularly flavonoid compounds, can vary depending on the rootstock, soil properties and environmental conditions where the trees are grown (Morales et al., 2020, 2021). In addition to citrus, this influence has also been observed in other woody and herbaceous species (Wulff et al., 1999; Yu et al., 2003; Liu et al., 2016). The influence of environmental conditions on the expression of a character such as FC and flavonoid contents makes the selection of a new grapefruit-like variety more difficult since these compounds need to be analyzed during several seasons, which requires additional efforts for breeding programs based on sexual hybridization. In this sense, identifying which strategy is the most appropriate for obtaining new varieties with low or no FC contents and enhanced flavonoid profiles will allow us to focus our efforts on the development of more efficient and sustainable citrus breeding programs.

### The Effect of Ploidy Level on Furanocoumarin and Flavonoid Content

**FIGURE 4:**
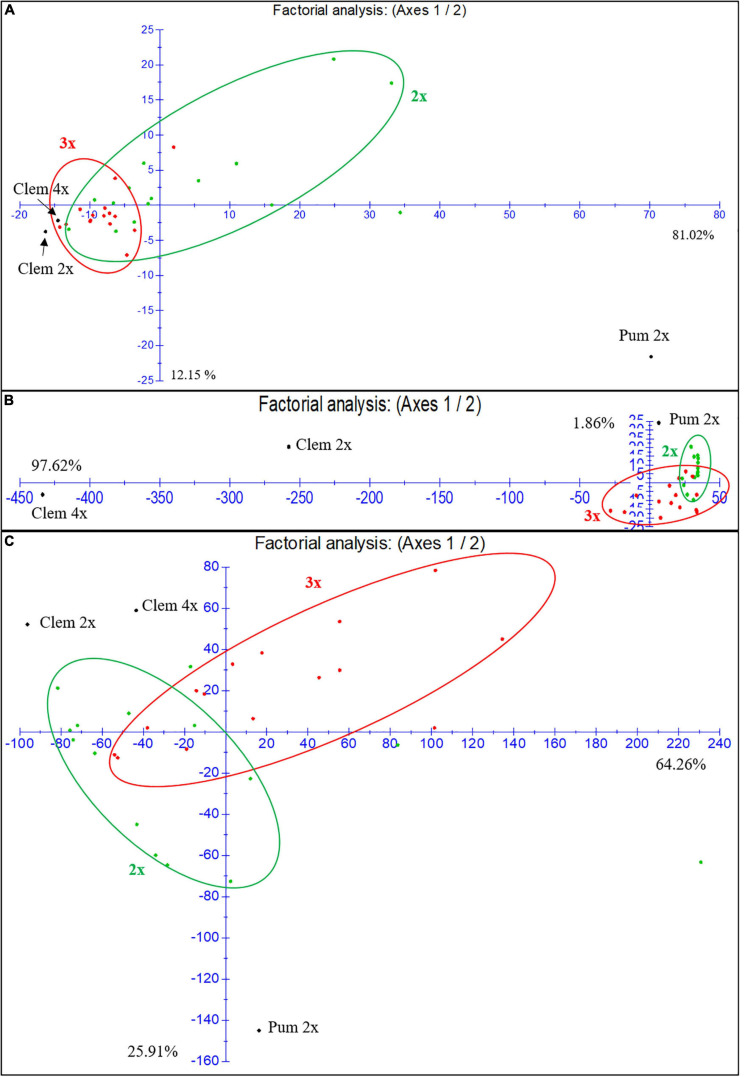
Factorial analysis of the FCs and flavonoid content for the three harvest times in the first season. **(A)** Furanocoumarins. **(B)** Narirutin and hesperidin flavonoids. **(C)** Naringin and neohesperidin flavonoids. 2x: diploid hybrids are colored green; 3x: triploid hybrids are colored red.

As we have indicated before, at the population level, triploid hybrids produce fewer FCs (6,7-DHB, bergapten and bergamottin) and more flavonoids such as eriocitrin, narirutin, hesperidin and particularly neohesperidin than diploid hybrids. In the factorial analysis of the FC and flavonoid contents for the three harvest times in the first season (Figure 4A), diploid and triploid hybrids have a closer FC profile to that of Clem 2x and 4x than to that of “Pink” pummelo. However, triploid hybrids were organized in a compact cluster with few exceptions, whereas diploid hybrids did not present a well-defined group displaying a dispersed distribution, indicating that at the population level, the FC profile of triploid hybrids is closer to the clementines FC profile, whereas diploid hybrids showed greater variability. These differences could be related to the double allele dosage received from the clementine genome, which is characterized by null or extremely low FC contents.

For the flavonoid content (Figures 4B,C), we focused the analysis on the most highly accumulated compounds (narirutin, hesperidin, naringin and neohesperidin). Figure 4B shows the analysis of narirutin and hesperidin, typical mandarin-like compounds, where we observed a clear influence (97.62%) of Clem 4x on the accumulation of these compounds in triploid hybrids. Most of the triploid hybrids are under axis one. Diploid hybrids were influenced by Pum 2x and Clem 2x, many of which are above axis one. As shown in Figure 4C, both diploid and triploid hybrids showed a more dispersed naringin and neohesperidin accumulation than FC accumulation, although there are more triploid hybrids above axis one than diploids, which means these triploids are closer to the female parents in this regard (Clem 2x and 4x). These results indicate that at the population level the narirutin and hesperidin contents of triploid hybrids are clearly influenced by the tetraploid clementine, whereas those of diploid hybrids are influenced by both diploid parents. Regarding the other flavonoid compounds, diploid and triploid hybrids showed great variability, although triploid hybrids had a profile closer to that of clementines than to that of pummelo. In summary, both diploid and triploid hybrids display greater variability in their profiles for flavonoids than for FCs.

As we indicated previously, ploidy level had an effect on FC and flavonoid composition of hybrids. To shed light on this statement, we additionally analyzed the FC and flavonoid contents of four commercial grapefruit varieties (“Flame”, “Rio Red”, “Star Ruby” and “Duncan”) at the diploid and tetraploid levels (Supplementary Table S1). Autotetraploid plants were recovered from spontaneous duplication of chromosomes in nucellar cells, which is a relatively frequent event in apomictic citrus genotypes such as grapefruit varieties (Aleza et al., 2011). Statistical differences were observed for the average values of the three furanocoumarins analyzed. 6,7-DHB was the furanocoumarin detected in the highest quantities, followed by bergamottin and bergapten. Tetraploid grapefruits produced less 6,7-DHB and bergamottin than diploids, whereas 4x grapefruits produced slightly more bergapten than diploids. The greatest difference between 2x and 4x grapefruits was observed for 6,7-DHB, with a maximum variation of 16.8 mg/L, although there was an important fluctuation between seasons. Tetraploid grapefruits also had lower FCs than Pum 2x, whereas Pum 2x and grapefruits produced similar amounts of FCs. All the grapefruits analyzed had a higher level of FCs than clementines, as reported by Dugrand-Judek et al. (2015). Regarding flavonoids (Supplementary Table S2), 4x grapefruits produced significantly higher amounts of narirutin, naringin, hesperidin and neohesperidin than 2x grapefruits for the three seasons analyzed. Moreover, for narirutin, naringin, and neohesperidin, 4x grapefruits had higher concentrations than the parents used in this study. In addition, differences were also observed between harvest times and seasons, as we indicated before, suggesting the influence of environmental factors on the concentration of these compounds. Chen et al. (2011) analyzed the FC content (bergamottin, 6,7-DHB and paradisin C) in only one season of diploid and triploid hybrids recovered by sexual hybridizations between diploid pummelos as female parents and diploid and tetraploid grapefruits and two allotetraploid somatic hybrids between sweet orange + pummelo as male parents. The authors also observed that at the population level, triploid hybrids produced less FC than diploid hybrids, and the FC concentrations generally changed irregularly with harvest time. All these results suggest that ploidy level has a particularly important influence on gene expression and regulation of FCs and flavonoid pathways.

Few studies have been performed in citrus to assess the effect of ploidy level on phenotypic variation (Cameron and Frost, 1968; Jaskani et al., 2002). Recently, Ahmed et al. (2020) have demonstrated that triploid mandarin hybrid progenies positively affected juice weight, juice percentage and sugar content in comparison with the diploid hybrids recovered with the same parents and they argued that genomic dosage effect could be the explanations for these variations. Polyploidy originates from new genomic configurations that are associated with diverse reorganizations and modifications affecting the structure and regulation of the new hybrid genome (Soltis and Soltis, 2009; Flagel and Wendel, 2010). These changes modify gene expression (Comai, 2005), and they may confer genome plasticity to improve the adaptation of polyploids to the environment (Chen, 2007). The new regulation of the parental genomes that constitute polyploid plants would explain the obtention of genotypes and phenotypes absent in the diploid pool (Osborn et al., 2003) and the non-additive inheritance (Chen, 2007; Bassene et al., 2009; Flagel and Wendel, 2010).

### Breeding Programs for the Selection of New Grapefruit-Like Varieties

Chen et al. (2011) stated that diploid and triploid hybrids appeared to cosegregate at an approximately 1:1 rate, with low and high concentrations of FCs, which suggests that biosynthesis of these FCs (6,7-DHB, bergamottin and paradisin C) could be controlled by a single enzymatic or regulatory gene. However, this segregation ratio was not observed in our work during the three different seasons.

Some attempts have already been made in different breeding programs worldwide to recover of new grapefruit-like citrus varieties with low FC contents. Fidel et al. (2016) reported the selection of two low FCs and seedless grapefruit-like varieties, named Aliza and Coocki, recovered by a cross between pummelo and mandarin. Fruits of these varieties resemble orange-fleshed grapefruits, and contain high levels of the flavanone naringin and only trace amounts of FCs. At the University of Florida, Gmitter (2015); US Plant Patent USPP26, 177P3) developed the UF-914 triploid hybrid between Siamese Sweet Acidless pummelo and tetraploid Ruby Red grapefruit (Grosser et al., 2015). This variety is characterized by red-fleshed, seedless, and relatively large fruit, with extremely low levels of FC. In Catania (Italy), a new grapefruit-like variety has been obtained in a cross between Monreal clementine and grapefruit, producing fruits with high juice and sugar content, slight bitter aftertaste, orange-flesh, and FC content ten times lower than that of commercial Marsh grapefruit (Russo et al., 2016). In our triploid breeding program (Navarro et al., 2015), we selected two new triploid grapefruit-like hybrids, IVIA TRI-750 and IVIA TRI-751, recovered in a cross between Fina clementine and tetraploid Duncan grapefruit, characterized by their high juice content, orange-flesh, and seedless fruits with very low FC levels. Currently, these varieties are being evaluated in collaboration with different Spanish citrus companies. In this way, the new grapefruit-like varieties with typical grapefruit attributes and orange pulp could be considered a new category of grapefruit, since present commercial grapefruit varieties are white, pink or red-fleshed, and no orange-fleshed category has been recognized until now.

## Conclusion

The strategy used for the production of new grapefruit-like citrus varieties has great impact on the amounts of FCs and flavanones that will subsequently be produced by the hybrids at the population level. Triploid hybrids recovered by Clem 4x by diploid “Pink” pummelo produce less bergapten, bergamottin and 6,7-DHB than diploid hybrids using the same parents but at the diploid level. Regarding flavonoids, differences have also been observed depending on each compound. Triploid hybrids produced more eriocitrin, narirutin, hesperidin and neohesperidin than diploid hybrids, whereas no differences were observed between hybrids in the neoeriocitrin and naringin contents. With these results we can conclude that at the population level and with the parentals used, the production of triploid hybrids by 4x × 2x sexual hybridizations is more appropriate than diploid hybrids recovery with diploid parents for the obtention of new grapefruit-like varieties with low FC and high flavonoid contents. Moreover, triploid hybrids do not produce seeds and do not induce the formation of seeds in other genotypes by cross-pollination, which is an essential characteristic for new citrus varieties (Cuenca et al., 2020). In addition, differences in the amount of these compounds have been observed between harvest times and seasons and could be associated with the effect of environmental conditions and maturation, which in practice makes the evaluation and selection of new varieties more difficult due to the need to quantify these compounds for each genotype in different months and seasons to avoid mistakes in the selection process and improve the efficiency of citrus breeding programs.

## Data Availability Statement

The original contributions presented in the study are included in the article/Supplementary Material, further inquiries can be directed to the corresponding author/s.

## Author Contributions

AG-L and PA conceived the study and were in charge of the direction and planning and took the lead writing the manuscript with input and review of LN and JC. AB and JM did the phenolic compounds extraction and the HPLC analyses and data collection. MH, AM, and JC did the fruit samples collection and juice extraction. All authors read and approved the final version of this manuscript.

## Conflict of Interest

The authors declare that the research was conducted in the absence of any commercial or financial relationships that could be construed as a potential conflict of interest.

## Supplementary Material

Supplementary Figure 1 Accumulation of chilling hours during the three harvest times, December, January and February, across the three seasons (2015/2016, 2016/2017, and 2017/2018). Maximum, medium, and minimum temperatures are represented on the left axis.

Supplementary Table 1 Average furanocoumarin concentration (mg/L) in the 4x and 2x grapefruits for three harvest times in the three seasons analyzed.

Supplementary Table 2 Average flavonoid concentration (mg/L) in the 4x and 2x grapefruits for three harvest times in the three seasons analyzed.
