# Supplementary material for: Strategies to Produce Grapefruit-Like Citrus Varieties With a Low Furanocoumarin Content and Distinctive Flavonoid Profiles
Source: Front Plant Sci. 2021 Feb 24;12:640512. doi: 10.3389/fpls.2021.640512 (PMC7943927; doi:10.3389/fpls.2021.640512)
Supplement: Supplementary Figure 1 — Accumulation of chilling hours during the three harvest times, December, January and February, across the three seasons (2015/2016, 2016/2017, and 2017/2018). Maximum, medium, and minimum temperatures are represented on the left axis. [file Presentation_1.PPTX]

## Slide 1
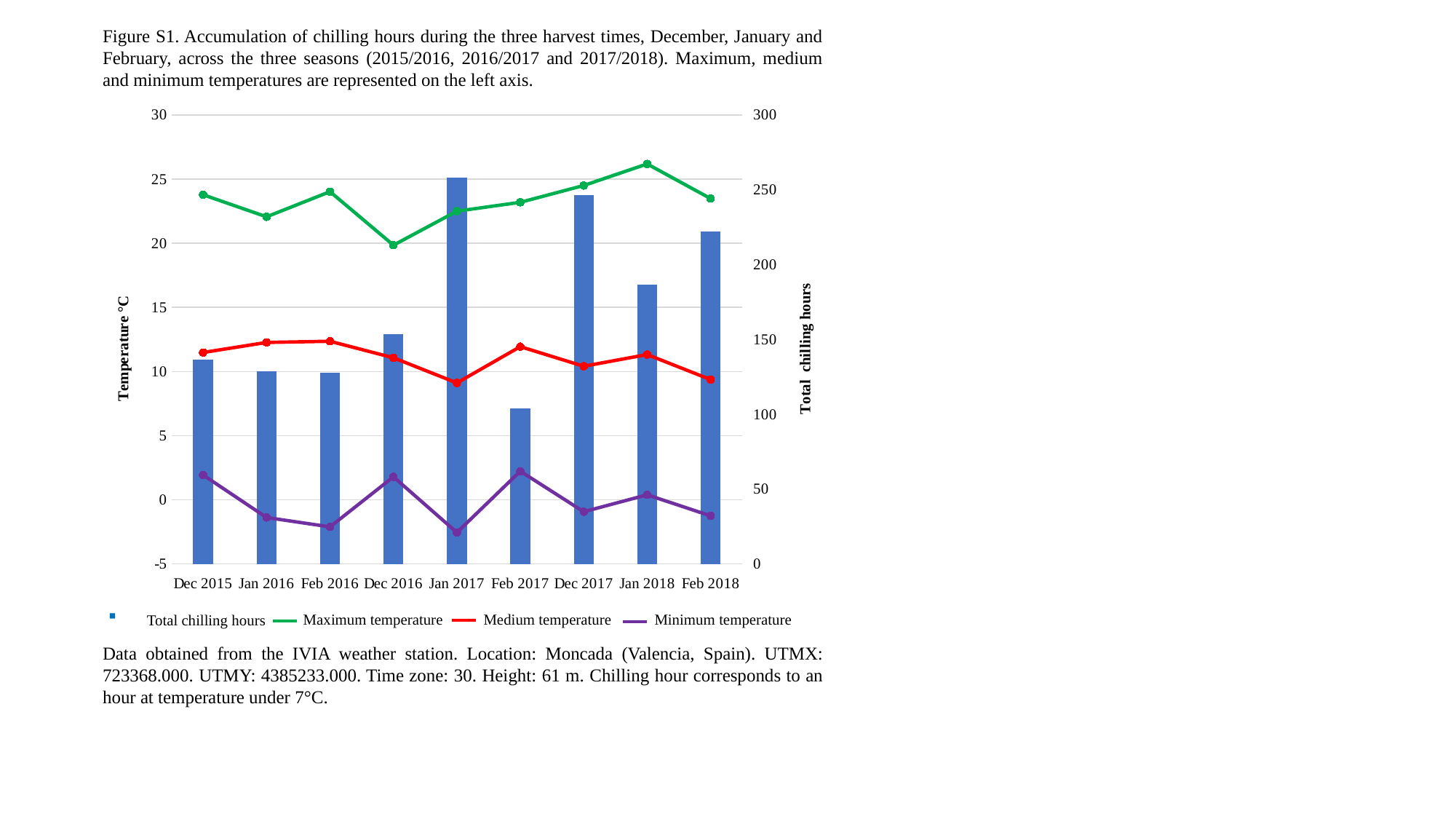

Figure S1. Accumulation of chilling hours during the three harvest times, December, January and February, across the three seasons (2015/2016, 2016/2017 and 2017/2018). Maximum, medium and minimum temperatures are represented on the left axis.
### Chart
| Category | Horas frío | Temp media de las medias | Temp máxima de las máximas | Temp mínima de las mínimas |
|---|---|---|---|---| Total chilling hours
Minimum temperature
Medium temperature
Maximum temperature
Data obtained from the IVIA weather station. Location: Moncada (Valencia, Spain). UTMX: 723368.000. UTMY: 4385233.000. Time zone: 30. Height: 61 m. Chilling hour corresponds to an hour at temperature under 7°C.
