# Supplementary material for: Strategies to Produce Grapefruit-Like Citrus Varieties With a Low Furanocoumarin Content and Distinctive Flavonoid Profiles
Source: Front Plant Sci. 2021 Feb 24;12:640512. doi: 10.3389/fpls.2021.640512 (PMC7943927; doi:10.3389/fpls.2021.640512)
Supplement: Supplementary Table 1 — Average furanocoumarin concentration (mg/L) in the 4x and 2x grapefruits for three harvest times in the three seasons analyzed. [file Table_1.DOCX]

Table S1. Average furanocoumarin concentration (mg/L) in the 4x and 2x grapefruits for three harvest times in the three seasons analyzed.

|  |  | **Bergapten** | | | **6,7-DHB** | | | **Bergamottin** | | |
| --- | --- | --- | --- | --- | --- | --- | --- | --- | --- | --- |
|  | **Samples** | **S1** | **S2** | **S3** | **S1** | **S2** | **S3** | **S1** | **S2** | **S3** |
| **HT1** | **Flame 4x** | 2.00 | 0.00 | 0.40 | 32.47 | 16.00 | 16.67 | 1.80 | 2.07 | 0.80 |
|  | **Rio Red 4x** | 0.93 | 0.00 | 0.60 | 25.07 | 14.67 | 26.67 | 0.20 | 1.27 | 2.40 |
|  | **Star Ruby 4x** | 1.20 | 0.00 | 0.40 | 32.47 | 12.93 | 28.20 | 2.60 | 2.07 | 2.87 |
|  | **Duncan 4x** | 0.87 | 0.00 | 0.60 | 37.87 | 9.00 | 23.73 | 3.80 | 1.93 | 2.07 |
|  | **Flame 2x** | 1.60 | 0.00 | 0.53 | 41.40 | 36.67 | 46.40 | 4.20 | 4.80 | 3.87 |
|  | **Rio Red 2x** | 0.07 | 0.00 | 0.47 | 50.93 | 23.40 | 33.67 | 2.80 | 2.73 | 3.20 |
|  | **Star Ruby 2x** | 1.53 | 0.00 | 0.93 | 53.93 | 32.33 | 12.33 | 6.80 | 3.07 | 1.73 |
|  | **Duncan 2x** | 1.53 | 0.00 | 0.60 | 122.93 | 38.80 | 42.73 | 6.80 | 2.93 | 3.20 |
| **HT2** | **Flame 4x** | 2.53 | 0.00 | 2.20 | 55.40 | 25.30 | 26.27 | 2.53 | 1.60 | 0.00 |
|  | **Rio Red 4x** | 3.47 | 0.00 | 1.13 | 63.60 | 10.60 | 39.07 | 3.47 | 1.80 | 0.00 |
|  | **Star Ruby 4x** | 0.33 | 0.00 | 0.93 | 25.53 | 20.00 | 34.33 | 3.00 | 1.60 | 2.87 |
|  | **Duncan 4x** | 0.33 | 0.00 | 0.00 | 24.07 | 33.90 | 14.93 | 2.20 | 1.60 | 1.93 |
|  | **Flame 2x** | 0.20 | 0.00 | 0.13 | 37.07 | 42.70 | 20.87 | 4.07 | 3.60 | 2.73 |
|  | **Rio Red 2x** | 0.27 | 0.00 | 0.00 | 42.20 | 19.20 | 17.07 | 4.67 | 2.30 | 2.53 |
|  | **Star Ruby 2x** | 0.00 | 0.00 | 0.00 | 44.13 | 26.00 | 10.87 | 4.40 | 2.70 | 2.00 |
|  | **Duncan 2x** | 0.00 | 1.30 | 0.00 | 51.73 | 93.20 | 31.60 | 1.80 | 1.80 | 3.80 |
| **HT3** | **Flame 4x** | 0.33 | 0.00 | 4.27 | 20.60 | 25.30 | 36.67 | 2.47 | 2.20 | 0.00 |
|  | **Rio Red 4x** | 0.60 | 0.00 | 0.00 | 19.27 | 10.70 | 57.27 | 1.73 | 1.60 | 0.00 |
|  | **Star Ruby 4x** | 0.20 | 0.00 | 0.73 | 30.40 | 16.20 | 13.60 | 2.13 | 1.80 | 0.60 |
|  | **Duncan 4x** | 0.33 | 0.00 | 1.20 | 40.07 | 13.30 | 11.07 | 2.07 | 1.80 | 1.40 |
|  | **Flame 2x** | 0.00 | 0.00 | 0.00 | 44.27 | 22.40 | 27.87 | 3.00 | 2.60 | 3.13 |
|  | **Rio Red 2x** | 0.20 | 0.00 | 0.00 | 27.40 | 24.30 | 18.67 | 2.87 | 2.40 | 1.93 |
|  | **Star Ruby 2x** | 0.00 | 0.00 | 0.00 | 16.93 | 16.40 | 20.33 | 1.93 | 2.00 | 2.20 |
|  | **Duncan 2x** | 0.00 | - | 0.00 | 67.40 | - | 30.73 | 4.00 | - | 2.67 |
| **the three** | **4x** | **1.10 ^*^** | **0.00** | **1.04 ^*^** | **33.90 ^*^** | **17.33 ^*^** | **27.37** | **2.33 ^*^** | **1.78 ^*^** | **1.24 ^*^** |
| **HT** | **2x** | **0.45** | **0.12** | **0.22** | **50.03** | **34.13** | **26.09** | **3.94** | **2.81** | **2.75** |

S1: Season 2015/2016; S2: Season 2016/2017; S3: Season 2017/2018; HT1: Harvest time 1, December; HT2: Harvest time 2, January; HT3: Harvest time 3, February.

Bold numbers indicate the average value of the three harvest dates for each season.

* Significant differences (p<0.005) between tetraploid and diploid grapefruits for each compound.

(-) No fruit available.
