# Supplementary material for: Strategies to Produce Grapefruit-Like Citrus Varieties With a Low Furanocoumarin Content and Distinctive Flavonoid Profiles
Source: Front Plant Sci. 2021 Feb 24;12:640512. doi: 10.3389/fpls.2021.640512 (PMC7943927; doi:10.3389/fpls.2021.640512)
Supplement: Supplementary Table 2 — Average flavonoid concentration (mg/L) in the 4x and 2x grapefruits for three harvest times in the three seasons analyzed. [file Table_2.DOCX]

Table S2. Average flavonoid concentration (mg/L) in the 4x and 2x grapefruits for three harvest times in the three seasons analyzed.

|  |  | **Eriocitrin** | | | **Neoeriocitrin** | | | **Narirutin** | | | **Naringin** | | | **Hesperidin** | | | **Neohesperidin** | | |
| --- | --- | --- | --- | --- | --- | --- | --- | --- | --- | --- | --- | --- | --- | --- | --- | --- | --- | --- | --- |
|  | **Samples** | **S1** | **S2** | **S3** | **S1** | **S2** | **S3** | **S1** | **S2** | **S3** | **S1** | **S2** | **S3** | **S1** | **S2** | **S3** | **S1** | **S2** | **S3** |
| **HT1** | **Flame 4x** | 0.00 | 0.00 | 0.00 | 0.00 | 4.57 | 0.00 | 68.60 | 190.00 | 190.47 | 376.27 | 1589.40 | 2010.13 | nd | 11.80 | 4.60 | 0.00 | 16.47 | 26.13 |
|  | **Rio Red 4x** | 0.00 | 0.00 | 0.00 | 0.00 | 10.53 | 0.00 | 157.67 | 207.73 | 246.60 | 1894.93 | 2180.47 | 1453.13 | nd | 20.47 | 13.20 | 14.67 | 21.13 | 29.33 |
|  | **Star Ruby 4x** | 0.00 | 0.00 | 0.00 | 0.00 | 9.27 | 0.00 | 97.40 | 181.80 | 162.93 | 851.13 | 2228.40 | 1788.47 | nd | 19.40 | 6.13 | 14.47 | 30.33 | 24.73 |
|  | **Duncan 4x** | 0.00 | 0.00 | 4.80 | 6.27 | 13.93 | 10.27 | 81.20 | 112.33 | 89.20 | 617.20 | 1815.00 | 466.13 | nd | 13.27 | 1.73 | 17.80 | 15.27 | 11.00 |
|  | **Flame 2x** | 0.00 | 0.00 | 0.00 | 4.20 | 4.40 | 0.00 | 52.40 | 58.33 | 49.80 | 243.73 | 242.07 | 523.33 | nd | 0.00 | 0.27 | 4.33 | 5.00 | 1.87 |
|  | **Rio Red 2x** | 0.00 | 0.00 | 0.93 | 4.40 | 5.20 | 1.13 | 42.40 | 50.47 | 61.87 | 198.13 | 199.80 | 263.93 | nd | 0.00 | 0.93 | 4.60 | 5.53 | 8.73 |
|  | **Star Ruby 2x** | 0.00 | 0.00 | 4.93 | 4.33 | 4.00 | 4.60 | 40.93 | 58.13 | 85.73 | 218.00 | 206.07 | 359.13 | nd | 1.73 | 6.53 | 5.27 | 6.53 | 32.73 |
|  | **Duncan 2x** | 0.00 | 0.00 | 0.80 | 4.53 | 6.13 | 1.00 | 44.80 | 62.27 | 40.60 | 200.53 | 348.27 | 196.33 | nd | 0.00 | 0.00 | 4.40 | 5.47 | 5.73 |
| **HT2** | **Flame 4x** | 0.00 | 0.00 | 0.00 | 0.60 | 0.00 | 0.00 | 90.47 | 258.90 | 274.13 | 764.80 | 2421.20 | 951.93 | 1.93 | 14.60 | 6.93 | 9.13 | 20.70 | 27.67 |
|  | **Rio Red 4x** | 0.00 | 0.00 | 0.00 | 0.60 | 0.00 | 0.00 | 94.13 | 223.80 | 203.00 | 802.60 | 2235.10 | 1338.73 | 0.93 | 19.90 | 48.80 | 11.27 | 20.80 | 28.07 |
|  | **Star Ruby 4x** | 0.00 | 0.00 | 0.00 | 0.00 | 0.00 | 0.00 | 57.80 | 206.40 | 152.73 | 575.73 | 2599.30 | 1415.60 | 0.20 | 19.50 | 1.73 | 11.20 | 23.50 | 25.07 |
|  | **Duncan 4x** | 0.00 | 0.00 | 1.33 | 4.00 | 0.00 | 5.13 | 97.33 | 160.60 | 56.87 | 749.40 | 1879.30 | 285.60 | 3.87 | 7.30 | 0.00 | 20.40 | 13.30 | 3.20 |
|  | **Flame 2x** | 0.00 | 0.00 | 1.67 | 2.40 | 0.00 | 2.07 | 39.47 | 54.90 | 47.27 | 228.00 | 148.40 | 163.73 | 2.07 | 0.00 | 0.27 | 4.93 | 3.00 | 3.60 |
|  | **Rio Red 2x** | 0.00 | 0.00 | 1.20 | 0.00 | 0.00 | 2.07 | 37.40 | 64.40 | 32.93 | 214.13 | 225.20 | 130.73 | 1.00 | 0.00 | 0.00 | 6.40 | 5.60 | 2.20 |
|  | **Star Ruby 2x** | 0.00 | 0.00 | 0.00 | 2.40 | 0.00 | 0.00 | 52.53 | 61.40 | 53.27 | 280.80 | 260.40 | 257.07 | 1.73 | 0.00 | 0.07 | 9.20 | 7.00 | 10.53 |
|  | **Duncan 2x** | 0.00 | 0.00 | 1.20 | 0.00 | 0.00 | 2.00 | 53.13 | 79.70 | 37.20 | 293.20 | 356.10 | 83.13 | 1.80 | 0.00 | 0.07 | 0.60 | 5.50 | 1.60 |
| **HT3** | **Flame 4x** | 0.00 | 0.00 | 0.00 | 10.27 | 0.00 | 0.00 | 103.13 | 229.00 | 287.27 | 1392.40 | 2594.20 | 2974.87 | 10.47 | 29.80 | 35.13 | 11.27 | 21.20 | 31.07 |
|  | **Rio Red 4x** | 0.00 | 0.00 | 0.00 | 0.00 | 0.00 | 0.00 | 122.07 | 212.90 | 258.67 | 1561.80 | 1519.80 | 3768.73 | 6.00 | 15.90 | 57.33 | 13.60 | 39.50 | 28.60 |
|  | **Star Ruby 4x** | 0.00 | 0.00 | 0.00 | 6.27 | 0.00 | 0.00 | 61.13 | 163.70 | 93.33 | 429.47 | 2490.30 | 834.20 | 2.53 | 14.20 | 0.33 | 9.87 | 39.80 | 16.67 |
|  | **Duncan 4x** | 0.00 | 0.00 | 0.00 | 6.53 | 0.00 | 0.00 | 73.67 | 125.80 | 37.93 | 727.80 | 2139.60 | 171.27 | 1.60 | 6.00 | 10.00 | 7.60 | 18.20 | 14.13 |
|  | **Flame 2x** | 0.00 | 0.00 | 0.00 | 5.40 | 6.50 | 0.00 | 43.60 | 66.20 | 50.60 | 272.07 | 382.70 | 201.33 | 0.33 | 2.30 | 3.53 | 3.80 | 13.90 | 7.00 |
|  | **Rio Red 2x** | 0.00 | 0.00 | 0.00 | 5.73 | 8.50 | 0.00 | 47.00 | 69.70 | 42.00 | 211.87 | 297.20 | 201.27 | 1.40 | 4.40 | 0.80 | 5.27 | 14.00 | 8.80 |
|  | **Star Ruby 2x** | 0.00 | 0.00 | 0.00 | 5.00 | 4.20 | 0.00 | 54.40 | 37.60 | 38.07 | 327.07 | 130.60 | 214.33 | 1.40 | 0.00 | 22.67 | 6.60 | 4.20 | 9.67 |
|  | **Duncan 2x** | 0.00 | - | 0.87 | 5.40 | - | 0.93 | 63.40 | - | 23.47 | 354.00 | - | 127.20 | 0.33 | - | 0.73 | 5.93 | - | 4.27 |
| **the three** | **4x** | **0.00** | **0.00** | **0.56** | **2.88** | **3.19** | **1.40** | **92.05 ^*^** | **189.41 ^*^** | **171.09 ^*^** | **895.29 ^*^** | **2141.01 ^*^** | **1454.90 ^*^** | **3.44 ^*^** | **16.01 ^*^** | **15.49 ^*^** | **11.77 ^*^** | **23.35 ^*^** | **22.14 ^*^** |
| **HT** | **2x** | **0.00** | **0.00** | **0.97** | **3.65** | **3.54** | **1.15** | **47.62** | **60.28** | **46.90** | **253.46** | **254.25** | **226.79** | **1.26** | **0.77** | **2.99** | **5.11** | **6.88** | **8.06** |

S1: Season 2015/2016; S2: Season 2016/2017; S3: Season 2017/2018; HT1: Harvest time 1, December; HT2: Harvest time 2, January; HT3: Harvest time 3, February.

Bold numbers indicate the average value of the three harvest dates for each season.

* Significant differences (p<0.005) between tetraploid and diploid grapefruits for each compound.

(nd) not detected.

(-) No fruit available.
